# Evaluating Bayesian spatial methods for modelling species distributions with clumped and restricted occurrence data

**DOI:** 10.1371/journal.pone.0187602

**Published:** 2017-11-30

**Authors:** David W. Redding, Tim C. D. Lucas, Tim M. Blackburn, Kate E. Jones

**Affiliations:** 1 Centre for Biodiversity and Environment Research, Department of Genetics, Evolution and Environment, University College London, London, United Kingdom; 2 Big Data Institute, University of Oxford, Oxford, United Kingdom; 3 Institute of Zoology, Zoological Society of London, London, United Kingdom; Universita degli Studi di Napoli Federico II, ITALY

## Abstract

Statistical approaches for inferring the spatial distribution of taxa (Species Distribution Models, SDMs) commonly rely on available occurrence data, which is often clumped and geographically restricted. Although available SDM methods address some of these factors, they could be more directly and accurately modelled using a spatially-explicit approach. Software to fit models with spatial autocorrelation parameters in SDMs are now widely available, but whether such approaches for inferring SDMs aid predictions compared to other methodologies is unknown. Here, within a simulated environment using 1000 generated species’ ranges, we compared the performance of two commonly used non-spatial SDM methods (Maximum Entropy Modelling, MAXENT and boosted regression trees, BRT), to a spatial Bayesian SDM method (fitted using R-INLA), when the underlying data exhibit varying combinations of clumping and geographic restriction. Finally, we tested how any recommended methodological settings designed to account for spatially non-random patterns in the data impact inference. Spatial Bayesian SDM method was the most consistently accurate method, being in the top 2 most accurate methods in 7 out of 8 data sampling scenarios. Within high-coverage sample datasets, all methods performed fairly similarly. When sampling points were randomly spread, BRT had a 1–3% greater accuracy over the other methods and when samples were clumped, the spatial Bayesian SDM method had a 4%-8% better AUC score. Alternatively, when sampling points were restricted to a small section of the true range all methods were on average 10–12% less accurate, with greater variation among the methods. Model inference under the recommended settings to account for autocorrelation was not impacted by clumping or restriction of data, except for the complexity of the spatial regression term in the spatial Bayesian model. Methods, such as those made available by R-INLA, can be successfully used to account for spatial autocorrelation in an SDM context and, by taking account of random effects, produce outputs that can better elucidate the role of covariates in predicting species occurrence. Given that it is often unclear what the drivers are behind data clumping in an empirical occurrence dataset, or indeed how geographically restricted these data are, spatially-explicit Bayesian SDMs may be the better choice when modelling the spatial distribution of target species.

## Introduction

Development of quantitative methods to predict the spatial distribution of taxa from occurrence data is an active area of ecological research [1–3]. Understanding where organisms are geographically located is key for many reasons: conservation scientists, for example, require knowledge about threatened species’ distributions to prioritise management efforts [4, 5]. Alternatively, community ecologists need to know which species are likely to be present in the broader species pool to better understand the community assemblage process at a specific location [6, 7]. Within disease research, knowledge about pathogen distributions across a landscape can better inform understanding of spatial patterns of human disease risk [8, 9].

Statistical approaches for inferring the spatial distributions of taxa across landscapes are commonly termed ‘Species Distribution Modelling’ (SDM) or ‘Niche Modelling’. Rather than estimating niches as such, or looking to create models to better understand the causative process behind spatial distributions, in most cases these statistical approaches are used as a spatial interpolation across a region of interest to overcome incomplete sampling and predict the probability of presence/absence at all un-surveyed locations. SDMs commonly rely on regression techniques, which identify the correlative associations of species’ occurrence to a suite of explanatory and spatially extensive variables, e.g. temperature, altitude, and rainfall [1, 10].

Over the last decade there has been a significant uptake in methods that fit highly complex SDM models, for example using maximum-entropy based lasso regressions or boosted regression trees (BRT) approaches [11]. This has been driven by the ability of these methods to quickly select and infer models with limited user input and apparent high fit to the data, using e.g. ‘area under receiver operating curve (AUC) statistic [12]. The availability of bespoke software packages such as MAXENT [1] and the R package ‘dismo’ [13] have helped augment the methodological uptake, as these packages are computationally inexpensive, user-friendly, free-to-user and produce visually appealing outputs.

The ease of use of these packages, however, can also drive a method-agnostic (or ‘standard settings’) approach to analysis [2]. By default, highly complex, ‘black box’ approaches can select models that are over-fitted to the data such that any predictions partially reflect the sampling biases of input datasets [1, 10]. Various methodological configurations have been suggested to prevent such problems arising, including: reducing the complexity of the final model by increasing penalties on additional parameters [14], reducing the number of predictors [15], accounting for the spatial patterns of samples by using background points generated with a similar spatial structure [16–18], or reducing the spatial autocorrelation of the sampling points in the analyses [19–21]. A straightforward approach to control one aspect of this sampling bias, i.e. the non-random spatial patterning of samples, is to directly incorporate a spatially-structured random term into the underlying regression models. However, until recently, formally incorporating such a term into SDM frameworks commonly used for spatial interpolation of species occurrence by ecologists, required the use of complex code [21]. Integrated Nested Laplace Approximation (INLA) Bayesian methods for fitting models with spatial random effects have been recently implemented for R in the R-INLA package [22–26] and offer a highly flexible modelling environment, which can incorporate a variety of spatial, random effects into binomial, additive regression models. INLA methods analytically determine the posterior marginal distributions for parameters [27], which affords a large reduction in computational time compared to search based (e.g. MCMC) methods. Furthermore, R-INLA provides computationally fast approximations to the spatial, random effect, which have been shown to be effective in producing SDM-type spatial predictions [27].

Bayesian methods that remove the effects of spatial autocorrelation inferring SDMs have the potential to both improve and deteriorate true predictive ability [28] and it is unclear in what situations they might offer an improvement over non-spatial methods. Empirically collected samples are known often to be spatially biased due to anthropogenic drivers, such as: spatially heterogeneous reporting rates around areas with high numbers of observers and biases towards areas with active sampling programmes [29, 30], or, high disease detection rates where there are existing identification/diagnostic facilities [31]. However, some or all of the spatial patterning contained within any set of taxonomic samples could also be driven by the underlying suitability of the environment, rather than the impact of anthropogenic drivers, meaning the clumping of points itself contains important information and should not be discounted [32]. The preferential use of pseudo-absence points in SDMs as an attempt to overcome the lack of true absence data adds a further set of problems during inference [33], with a lack of clear framework to select appropriate sets of background points [34]. The computational packages that are most commonly used and easily applicable by non-expert audiences, however, are heavily weighted towards presence-ground approaches and we focus on them here, mindful that point-process models might offer a better way forward [18]. Here, we test the role that clumping and geographical bias have on the predictive ability of presence-background modelling methods, comparing a spatially-explicit Bayesian approach (spatially-explicit INLA) to three non-spatial methods (non-spatial INLA, MAXENT and BRT) on sets of simulated data that show high variation in clumping and bias. We also test, for all methods, if inferences using any of the best-practice user settings previously recommended for optimum SDM analysis are sensitive to our measured data biases. Overall, we show how the processes behind the spatial patterns of the input data can dictate the optimal choice of methods, showing that for the commonly encountered scenario of having clumped sample data, spatial Bayesian models are more consistently accurate than traditional methods.

## Methods

We tested the predictive performance of different SDM methods to reconstruct spatial presence, using simulated data sets with varying degrees of data clumping and geographical bias for 1000 hypothetical taxa. All analyses was performed using R [35]. For each taxon, we generated four sets of simulated data with which to reconstruct occurrence, as follows:

1. Covariate raster layers. We first generated 5 covariates (or ‘explanatory variables’ to represent the role of bio-climatic data layers such as temperature or rainfall) across a hypothetical landscape of 1-degree grid cells covering the world (a common resolution for SDM models and input data). We sampled from randomly generated Gaussian distributions to produce surfaces that best represent empirically observed climatic data. These surfaces, therefore, had left to right, top to bottom or diagonal gradients of values or had humped shaped sets of values, depending which part of the Gaussian distribution was sampled (e.g. the whole distribution or just one tail).
2. True presence raster layer. We employed the covariates in a presence-absence binomial regression, with randomly generated slopes and intercept, to calculate the true spatial distribution of each hypothetical taxon. The regression formula was generated using a random number of terms taken from a pool consisting of all linear and square terms for each covariate and first order interactions between each of the five terms, giving a total of 25 possible terms in the most complex models. We generated the ‘true presence’ grid by then predicting a surface using the generated regression model with the original covariate raster layers as inputs. After generating this layer, we added a small amount of random variation (i.e. noise) to each grid cell to create a more realistic problem, where exact covariate relationships are unknown (due to uncertainty in remotely-sensed data, for example).
3. Validation data. We sampled the true presence layer using 1000 random locations to create a validation data set of true presence and absence points, which we could use to evaluate the predictions from different SDM methods.
4. Spatially biased sample data. We then created a ‘samples’ data set with differing amounts of clumping and geographical bias, analogous to a typical input data used in an empirical SDM analysis. To create the biases, we first selected a small random number of ‘seed’ points from across the simulation landscape. These seed points were then filtered (i.e. removed or not) using a spatially heterogeneous sample reporting layer reflecting that, at some locations, external factors would alter the reporting rate of observations. This reporting rate varied randomly between 0 and 1 within six, randomly-sized and placed, but contiguous, areas across the landscape. We then generated the final SDM input dataset by randomly drawing a set of ‘sampling’ points from around each remaining ‘seed’ points, with a random clumping coefficient (the mean of a Gaussian distribution, ranging from 1–50 with a standard deviation equal to the mean divided by 5) dictating how tightly clustered any secondary sampling points were around each seed point. The number of ‘samples’ around each ‘seed’ was constrained to be either conditionally dependent on the probability of true presence or unaffected by the underlying suitability of the landscape. We term these two processes ‘biological’ bias and ‘random’, respectively. Therefore, for random datasets, sampling density was entirely random with respect to the underlying habitat suitability, representing the situation where any spatially heterogeneous sampling effort is driven by convenience or other non-biological processes. For biological bias, the density patterns in the spatially-biased samples were driven by the probability of the taxon’s true presence, representing the situation where a greater number of reports are made where there are more actual individuals to observe. All the secondary points (ranging between 25 and 500 points) were then used as the final sampling dataset to be brought forward to the SDM analysis.
5. Analysis. After simulation, we measured two aspects of the spatial pattern of the sample points in such a way that could be applied to a typical SDM input dataset, as follows: Clumping was defined as Clark-Evan’s dispersion coefficient of the samples [36] and split into high and low categories (‘clumped’ or ‘even’) by the median value; Geographical bias was calculated as the area covered by a convex hull containing all the biased samples, divided by the range of occurrence of the true positive (validation) samples, again split into high and low categories (‘high coverage’ or ‘restricted’) using the median value. This latter measure represents when the state of knowledge, for a given species, is solely the distributional limits of its geographical range of occurrence. Each dataset was then assigned to one of four equal groups, termed: even & high coverage, clumped & high coverage, even & restricted, and clumped & restricted.

For each taxon, we reconstructed the geographic range of occurrence using the simulated sampling data set and the covariate raster layers using the following SDM methods: (a) MAXENT [1]; (b) boosted regression trees BRT; (c) spatial Bayesian model [22–26]; and (d) non-spatial Bayesian model [22–26]. The MAXENT (a) and BRT (b) approaches are non-spatial methods commonly used in SDM analyses and been shown to have high predictive performance (11). To achieve this, MAXENT (a) fits a number of non-linear functions so that they explain the presence points while being uninformative over randomly selected background points. In contrast, BRT (b) contrasts presence points to randomly selected pseudo-absence points. It then fits many regression trees, up-weighting the importance of misclassified points for each new tree (boosting).

The spatial Bayesian model (c) involves fitting a Bayesian, hierarchical, binomial GLM, with a continuous-space random-field. While, the same model could be fitted with MCMC or other approaches, we focus here on the R-INLA implementation as its ease of use makes the model easily accessible to many ecologists. We therefore refer to the combination of the spatial, hierarchical model, the INLA approximation and the R-INLA implementation as the “spatially-explicit INLA model”. In R-INLA, the random-field is implemented using a Gaussian Markov random field approximation to the fully continuous Gaussian random field. The model is hierarchical in that hyperparameters governing the effective spatial range of the random-field are fitted jointly with the regression parameters. Defining the hyperparameters as a random variable in this way avoids unprincipled selection of a fixed hyperparameter value; a common problem in frequentist spatial models. The non-spatial INLA model (d) is simply a Bayesian binomial GLM fitted with R-INLA and was used to ascertain whether the performance of the spatially-explicit INLA model (c) was due to the spatial random-field or other aspects of the INLA model).

We then predicted the true state (i.e. present or absent) for each species, for each species, at each of the locations of the 1000 validation data points, and used an AUC (Area Under operating Curve score) approach to calculate the predictive accuracy of each method by comparing the validation data with the predicted presence value. While other methods are available, AUC represents a commonly used and adequately performing measure of predictive accuracy [37] and works by calculating the relative numbers of correctly and incorrectly identified predictions across all possible classification threshold values of the binomial response, with an AUC value equal to or below 0.5 indicating a predictive ability equal to random expectation and 1 a perfect predictive ability[12].

Finally, we tested whether any of the previously recommended configurations for setting up any of the methods were sensitive to clumping or geographical restriction of the data. We employed a brute-force approach, testing all reasonable SDM method configurations (S1 Table) and ensuring that we included those previously identified method setups shown to improve predictive accuracy with biased data.

For R code, see supplementary code in S1 File. We also provide an R package, INLAutils (https://github.com/timcdlucas/INLAutils), using functions for fitting SDM models with R-INLA and more general utility functions for working with R-INLA.

## Results

**Fig 1:**
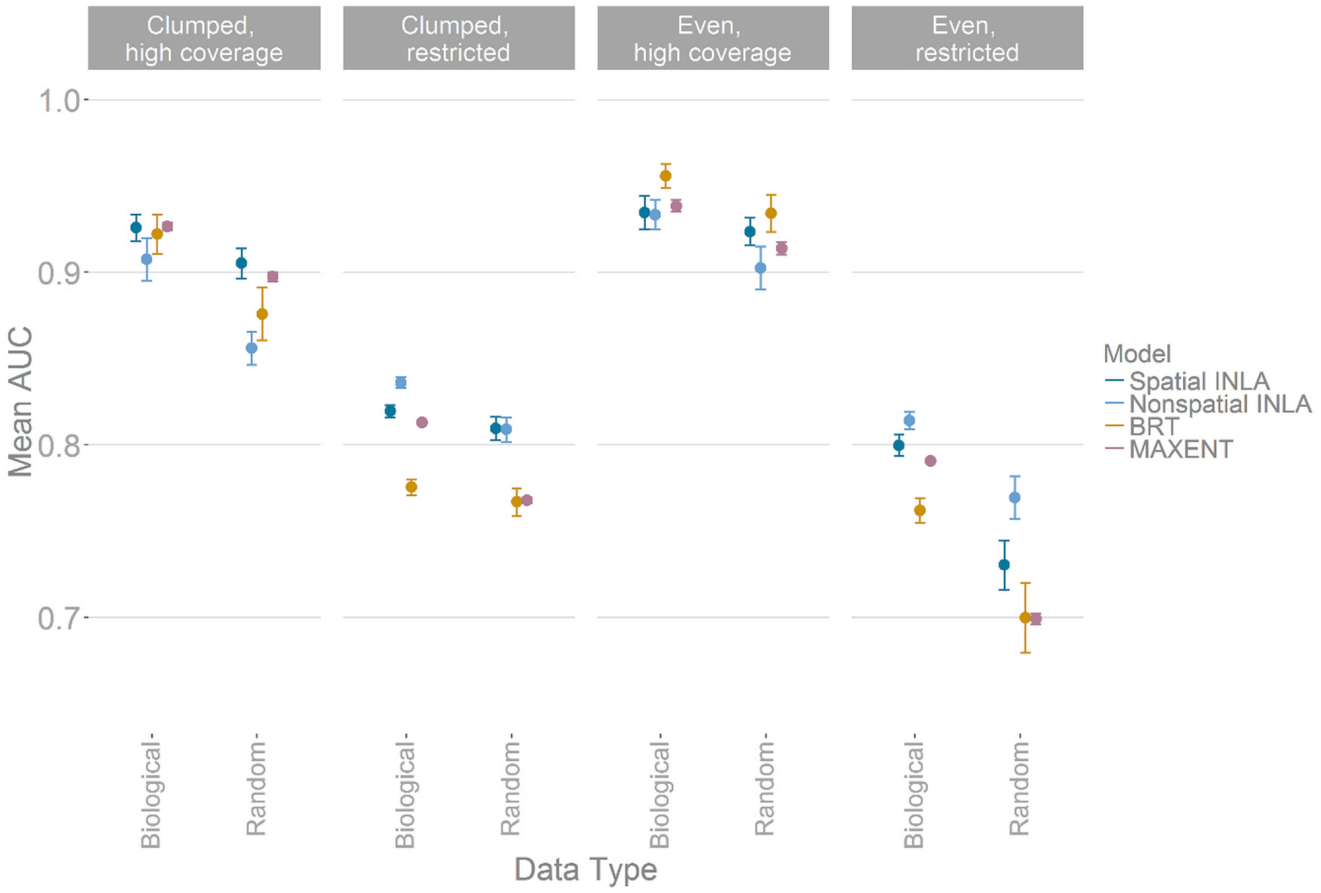
Comparison of the mean accuracy (AUC) of SDM models over 1000 simulated taxa. Sample clumping is caused by either biological or random processes. Panels show the predictive accuracy of data subsets binned into either high or low clumping and high or low coverage of the simulated true range. Points represent mean AUC scores from 1000 validation points per taxa and whiskers 95% confidence intervals around each mean, where scores less than 0.5 represent no accuracy gain over random chance. Spatial INLA—Bayesian SDM inferred using Integrated Nested Laplace Approximation with a spatial autocorrelation term, Non-spatial INLA—Bayesian SDM inferred using INLA without a spatial autocorrelation term, BRT—boosted regression trees based SDM, MAXENT—maximum entropy based SDM.

When comparing across sampling bias scenarios, the spatially-explicit INLA model was the most consistently accurate, being in the top two most accurate methods in 7 out of the 8 combinations examined here (Fig 1). The proportion of the simulated landscape covered by the sampling points was a key factor in dictating predictive accuracy, though presence of clumping did also confer a small loss in accuracy (Fig 1). Within the high coverage scenarios, when analysing datasets with low sample clumping (even & high coverage) all methods gave high predictive performance (Fig 1), but with BRT the most accurate (mean AUC 0.955 and 0.935 for biological and random clumping processes respectively), and with the non-spatial INLA model the least accurate (0.93 and 0.89 for biological and random clumping). For high coverage datasets with significant clumping of points (clumped & high coverage), the most accurate method was the spatially-explicit INLA model for both biological and random underlying processes (mean 0.929 and 0.901 AUC), again with the non-spatial INLA model performing the poorest (mean 0.914 and 0.865 AUC) (Fig 1).

Within low coverage sampling datasets (even & restricted and clumped & restricted), there were similar patterns among the methods, with an average low predictive accuracy compared to high coverage sampling data (0.71–0.84 AUC scores across all methods) and higher variance in scores. In both cases of restricted data, the simplest modelling approach, non-spatial INLA models, tended to perform better (Fig 1), with spatially-explicit INLA model the next most accurate method. When comparing clumping processes, if clumping was driven by biological processes rather than by random processes predictive accuracy was generally higher (Fig 1).

**Fig 2:**
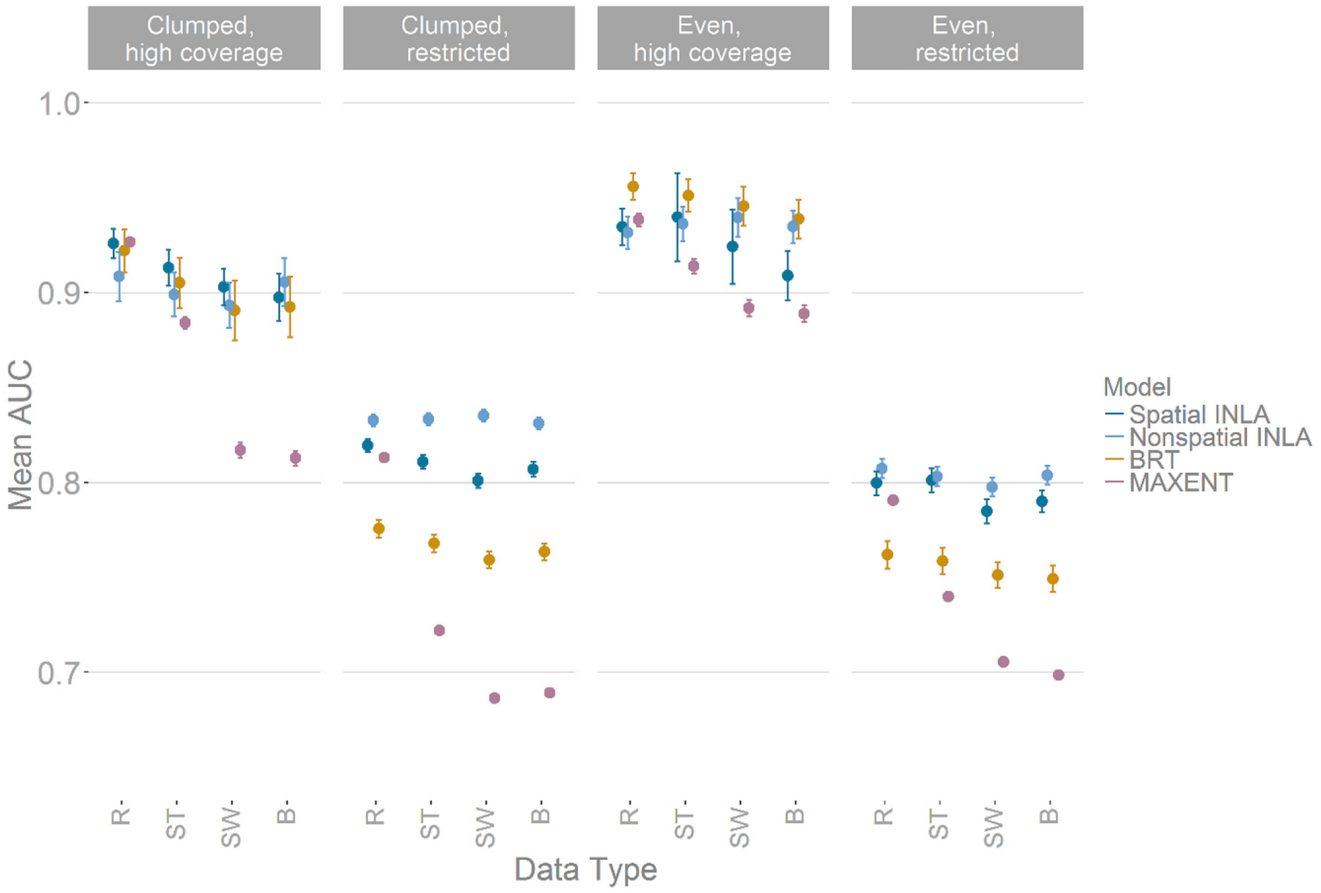
Comparison of the mean accuracy (AUC) of SDM models over 1000 simulated taxa when altering the pseudo-absence (background) point configurations and the effects of spatial thinning of presence points, on four SDM methods and across 4 types of dataset with different clumping and spatial bias. Panels show the predictive accuracy of data subsets binned into either high or low clumping and high or low coverage of the simulated true range. Where R represents random absence points, ST—spatial thinning, SW—spatially weighted absence points, B—both weighting and thinning (S1 Table) and Spatial INLA—Bayesian INLA model with spatial random effect, Non-spatial INLA—Bayesian INLA model without spatial autocorrelation term, BRT—boosted regression trees, and MAXENT—Maximum entropy based model. Points represent mean AUC scores from 1000 validation points per taxa and whiskers 95% confidence intervals around each mean, where scores less than 0.5 represent no accuracy gain over random chance.

For most methods, there was no difference in terms of predictive accuracy when choosing the best set-up of analysis options across clumped and restricted sampling groups. For instance, in all cases, randomly placed pseudo-absences/background points (R—S1 Table and Fig 2) out-performed both spatially-weighted absence points (SW) and spatially-thinned presence points (ST) in our analysis (Fig 2). Reducing the number of covariates always reduced average predictive accuracy (average reduction of 0.068 AUC score across methods) and including interaction terms in the formulas resulted in no significant gains in accuracy, irrespective of the complexity of the function used to generate the simulated data (S1 Fig).

**Fig 3:**
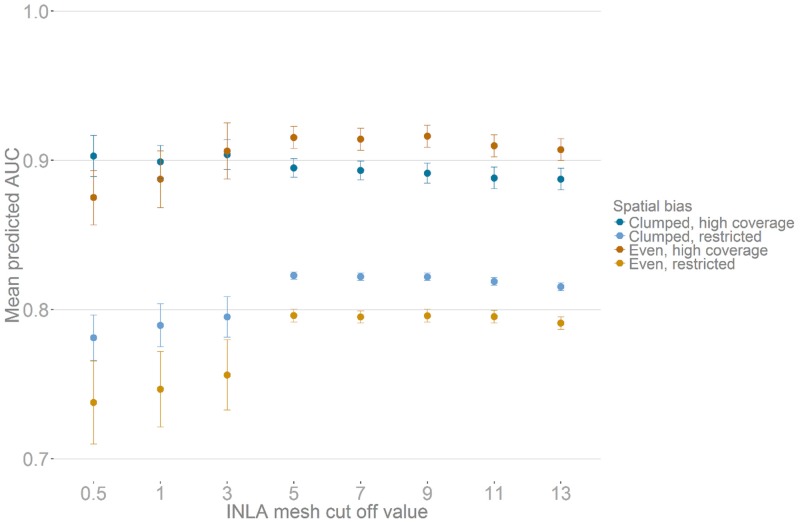
Comparison of mean accuracy (AUC) of spatially-explicit INLA SDM models on 1000 simulated taxa when varying the complexity of the underlying spatial mesh. Colours show the predictive accuracy of data subsets binned into either high or low clumping and high or low coverage of the simulated true range. Points represent mean AUC scores across 1000 taxa and whiskers 95% confidence intervals around each mean, where scores less than 0.5 represent no accuracy gain over random chance.

For INLA models comparing cut-off values from 0.5 to 8, we show that for high coverage-high clumping datasets (Fig 3) the smaller the cut off (and therefore the more complex the resulting spatial term), the more accurate the final models are. For data with low clustering and high coverage and with both low coverage datasets (Fig 3), there appears to be the opposite relationship with highest accuracy at values at a cut-off of around 3 to 6. For MAXENT models, increasing the regularisation (beta) setting, which preferentially selects less-complex models, generally resulted in less accurate results (S2 Fig). Also, when manually specifying model features, the inclusion of ‘hinge’ factors was important for good predictive accuracy (S3 Fig). None of the BRT initial set-up values (S4–S7 Figs) produced any clear difference in predictive accuracy.

## Discussion

Choosing the best method to undertake species distribution modelling depends on the spatial patterning within the input data. For instance, on even & high coverage datasets all methods performed well, especially boosted regression trees. Such datasets are likely to be infrequently found but when they are, the ease of use and high accuracy of MAXENT and BRT mean these methods make them ideal choices. For all types of clumped data, the spatially-explicit INLA model performed consistently well by controlling for the spatial pattern and therefore avoiding biases in the spatial distribution of data from causing biases in the inference of regression coefficients.

It is expected, and supported here, that clumping driven by underlying habitat suitability has less of an impact on the predictive performance of the models than random clumping, but, importantly, not by a large amount. In the case where SDMs are inferred using data sources such as GBIF, the complex processes underlying any spatial patterns in the input data are often unclear [16], and it appears that a precautionary approach of using a spatially-explicit method would reduce the impacts of misidentifying the processes behind underlying spatial bias. Sampling restriction, however, had a much larger impact on predictive accuracy than clumping. Building an SDM for a species based on sampling just one part of the overall range appears to risk biasing the underlying regression models. Here, the simple models produced by non-spatial INLA (and likely GLM approaches) appear to do well, perhaps because they are less over-fitted to any biases in the data, producing more general, less precise predictions.

Our results, therefore, show that it is important to remain sceptical about SDM predictions with high AUC scores that are based on a sub-sample of the input dataset, if there is no explicit measure of the proportion of the known range that has been sampled. With geographically restricted samples results with AUC scores >0.95 in our simulations often had a real AUC score of less than 0.75. Without taking into account the data sampling scenarios examined here, and instead evaluating SDM methods using the best-case datasets (i.e. even & high coverage), most analyses would prefer a BRT approach, which was a relatively poor performer on the clumped and restricted datasets.

In terms of evaluating previously recommended settings for each method, irrespective of the data sampling scenario, reducing the number of covariates to decrease collinearity was again shown to be an inefficient approach [15]. It seems that even variables with a minimal ability to explain the variation in species presence, confers a benefit greater than any cost arising from increasing the complexity of the model, though we note covariate collinearity could still obfuscate any attempted model interpretation. Conversely the role of changing pseudo-absence patterns away from random did not repeat the results of previous work with thinning [38] and spatial weighting [39] providing no clear gain in performance. The use of pseudo-absence (also called background) points is simply an ad-hoc [33] solution to the common problem of the lack of known non-occupied sites (i.e. input data contains only the recorded presence or individual sightings of target species) and there is a lack of clarity on the number and spatial spread of background points needed to improve predictive accuracy.

Some recent developments have focussed instead on log-Gaussian Cox process [40] and Poisson point process models (PPM) [18, 33] which, instead of using presence/absence data, use the spatial pattern of presence points as the dependent variable in a regression. Indeed, inferences using logistic type regressions with very large numbers of pseudo-absence points have slope values that tend towards that of similar Poisson point process model (PPM) [18]. With these presence-only modelling approaches, it is still unclear how they deal with specifically parsing biological and non-biological drivers of point density patterns. Spatial autocorrelation is known to be a problem for reliable inference of PPMs [34] but accounting for it when using MAXENT or R-INLA is complex and requires careful parameterisation [33]. Undoubtedly point-process models will soon become more user friendly and may prove a more effective approach but, in the meantime, it is important to evaluate the performance of the most commonly used approaches. An alternative, a non-regression based approach, “range bagging” [41] looks to bootstrap the variation in the multi-dimensional, environmental limits of a species, given random subsets of covariates and presence points, and careful evaluation of these methods is needed in respected to the performance of binomial regressions and PPMs.

The R-INLA package appears to offer additional benefits beyond spatially-explicit modelling. The combination of using a complex spatial latent field to capture spatial processes and an underlying simple additive regression model for the response variables relationship to environmental covariates, means that (specifically, when compared to boosted regression trees and lasso techniques) the fixed effects are potentially more straightforward to interpret [42] (i.e. per unit change in x results in per unit change in y). Another benefit of a Bayesian approach is the capturing of uncertainty for each predicted value, with predictive uncertainty an often ignored aspect of SDM modelling and prediction. R-INLA models are extremely flexible in their specification, with spatial autocorrelation and observer bias being straightforwardly incorporated as random effects, while standard error distributions, such as Gaussian, Poisson, binomial, and a variety of zero-inflated models, can be used interchangeably [22]. This method, therefore, has a built-in potential for extending SDM analysis away from simple binomial models by, for example, incorporating two or more types of data [43], hierarchical seasonal models [44] or fitting point-process models [33]. We hope that our study will aid the uptake of such fast spatial Bayesian methods, as this approach shows great promise for other analyses throughout ecology and evolutionary biology, especially in situations where non-independent samples are commonly experienced.

## Supporting information

S1 Fig Predictive accuracy of three species distribution modelling methods to infer 5000 simulated species’ ranges that were generated using either (a) just additive or (b) additive and interaction terms in formula used to determine the relationship between species’ presence and a set of simulated covariates. A repeated set of comparisons (c-d) is made for SDM methods (IN—spatial INLA, MAX—MAXENT, BRT—boosted regression trees) where interactions can also be specified for the inference formulae (i.e. INLA & MAXENT). Points represent mean AUC score over all simulated species where a prediction of the true range is attempted using a set of simulated sampling points, with whiskers showing the 95% confidence intervals. Different colours show the predictive accuracy of subsets of the 5000 datasets when binning the input samples from each dataset into either high or low clumping and high or low coverage of the simulated “true” range.
(TIF)

S2 Fig Predictive accuracy of the MAXENT species distribution modelling method when varying the complexity of the inference models using the beta, or “regularisation”, coefficient. Points represent mean AUC score over a set of 5000 simulated species where a prediction of the true range is attempted using a set of simulated sampling points, with whiskers showing the 95% confidence intervals. Different colours show the predictive accuracy of subsets of the 5000 datasets when binning the input samples from each dataset into either high or low clumping and high or low coverage of the simulated “true” range.
(TIF)

S3 Fig Predictive accuracy of the MAXENT species distribution modelling method when varying what class of terms are included in the inference model. Letter labels on x-axis represent model terms (Hinge—H, Product—P, Quadratic—Q, Threshold—T, Linear—L, Auto Feature—AF). Points represent mean AUC score over a set of 5000 simulated species where a prediction of the true range is attempted using a set of simulated sampling points, with whiskers showing the 95% confidence intervals. Different colours show the predictive accuracy of subsets of the 5000 datasets when binning the input samples from each dataset into either high or low clumping and high or low coverage of the simulated “true” range.
(TIF)

S4 Fig Predictive accuracy of boosted regression trees (BRT) species distribution modelling method when varying the learning rate of tree inference algorithm. Points represent mean AUC score over a set of 5000 simulated species where a prediction of the true range is attempted using a set of simulated sampling points, with whiskers showing the 95% confidence intervals. Different colours show the predictive accuracy of subsets of the 5000 datasets when binning the input samples from each dataset into either high or low clumping and high or low coverage of the simulated “true” range.
(TIF)

S5 Fig Predictive accuracy of boosted regression trees (BRT) species distribution modelling method when varying the complexity of the underlying regression trees during inference. Points represent mean AUC score over a set of 5000 simulated species where a prediction of the true range is attempted using a set of simulated sampling points, with whiskers showing the 95% confidence intervals. Different colours show the predictive accuracy of subsets of the 5000 datasets when binning the input samples from each dataset into either high or low clumping and high or low coverage of the simulated “true” range.
(TIF)

S6 Fig Predictive accuracy of boosted regression trees (BRT) species distribution modelling method when varying the bag fraction used to hold-back parts of data for internal validation. Points represent mean AUC score over a set of 5000 simulated species where a prediction of the true range is attempted using a set of simulated sampling points, with whiskers showing the 95% confidence intervals. Different colours show the predictive accuracy of subsets of the 5000 datasets when binning the input samples from each dataset into either high or low clumping and high or low coverage of the simulated “true” range.
(TIF)

S7 Fig Predictive accuracy of boosted regression trees (BRT) species distribution modelling method when varying the number of regression trees retained in the final modelling set. Points represent mean AUC score over a set of 5000 simulated species where a prediction of the true range is attempted using a set of simulated sampling points, with whiskers showing the 95% confidence intervals. Different colours show the predictive accuracy of subsets of the 5000 datasets when binning the input samples from each dataset into either high or low clumping and high or low coverage of the simulated “true” range.
(TIF)

S1 File R code used to run analysis. (ZIP)

S1 Table Options tested for four different species distribution model methods (for details see text). Letters in ‘Setting’ columns indicate abbreviations used and numbers previous analyses from the literature.
(DOCX)
