# Supplementary figures and images for: Evaluating Bayesian spatial methods for modelling species distributions with clumped and restricted occurrence data
Source: PLoS One. 2017 Nov 30;12(11):e0187602. doi: 10.1371/journal.pone.0187602 (PMC5708625; doi:10.1371/journal.pone.0187602)

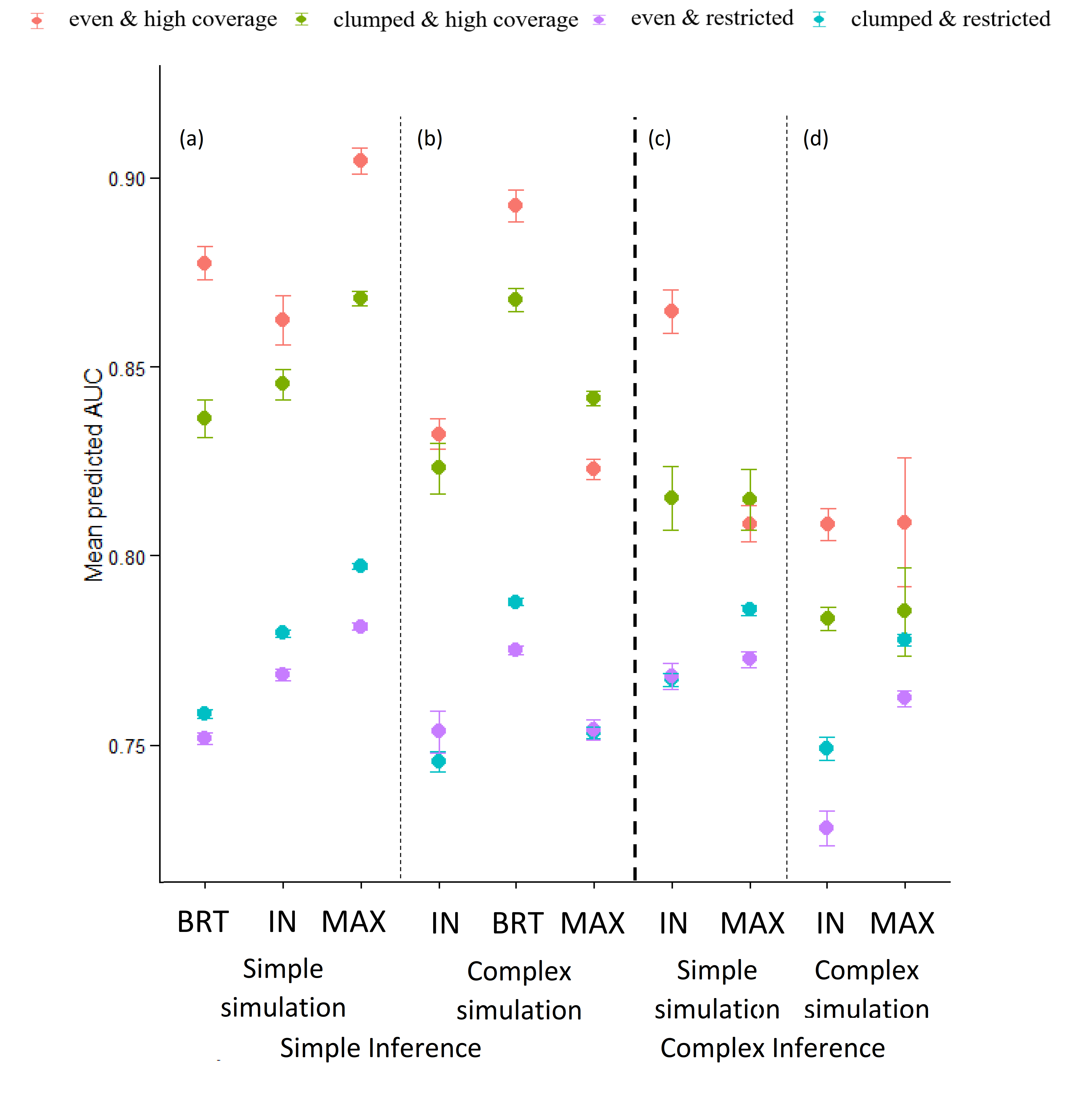

Supplement: S1 Fig — A repeated set of comparisons (c-d) is made for SDM methods (IN—spatial INLA, MAX—MAXENT, BRT—boosted regression trees) where interactions can also be specified for the inference formulae (i.e. INLA & MAXENT). Points represent mean AUC score over all simulated species where a prediction of the true range is attempted using a set of simulated sampling points, with whiskers showing the 95% confidence intervals. Different colours show the predictive accuracy of subsets of the 5000 datasets when binning the input samples from each dataset into either high or low clumping and high or low coverage of the simulated “true” range. (TIF) [file pone.0187602.s001.tif]

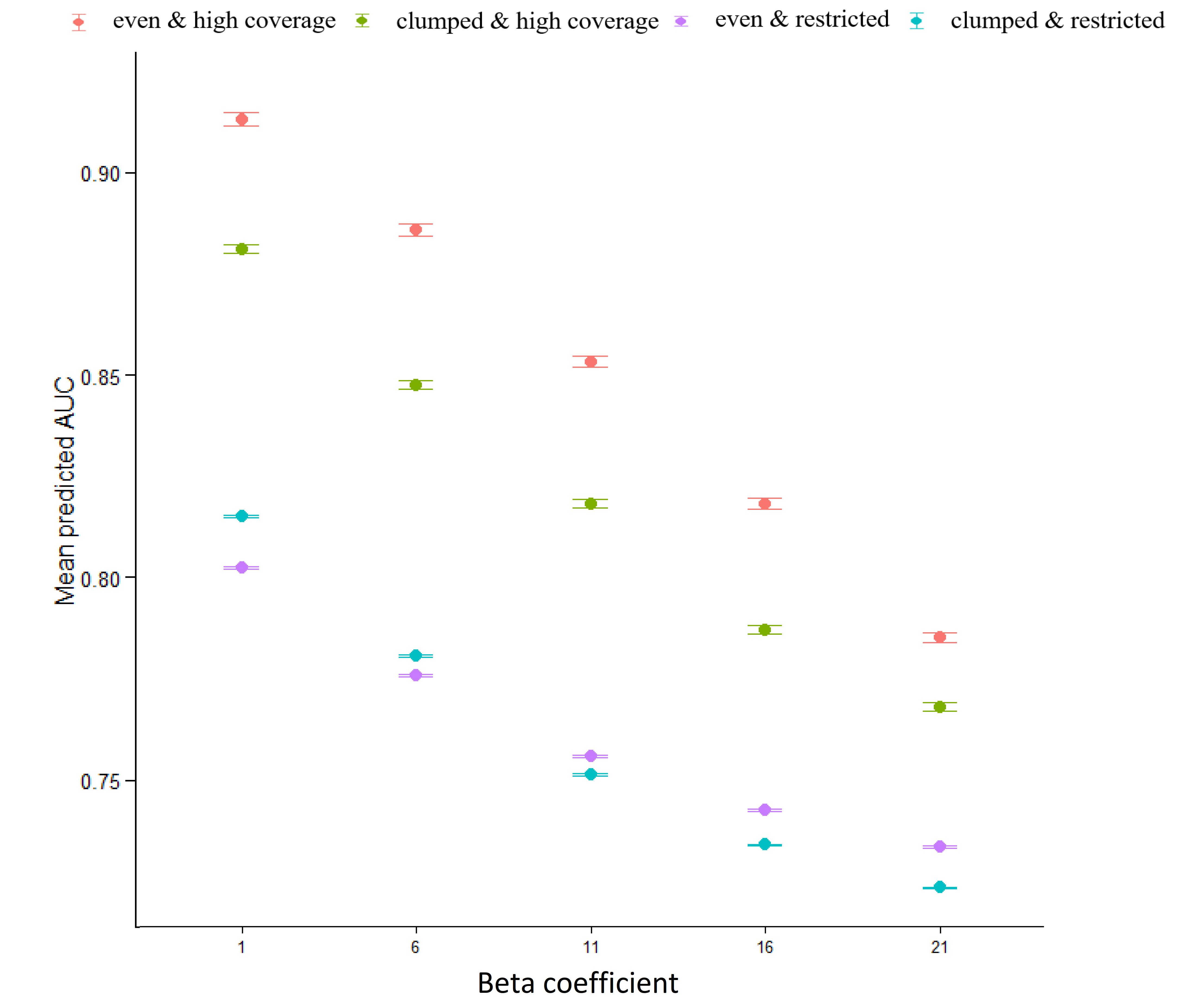

Supplement: S2 Fig — Points represent mean AUC score over a set of 5000 simulated species where a prediction of the true range is attempted using a set of simulated sampling points, with whiskers showing the 95% confidence intervals. Different colours show the predictive accuracy of subsets of the 5000 datasets when binning the input samples from each dataset into either high or low clumping and high or low coverage of the simulated “true” range. (TIF) [file pone.0187602.s002.tif]

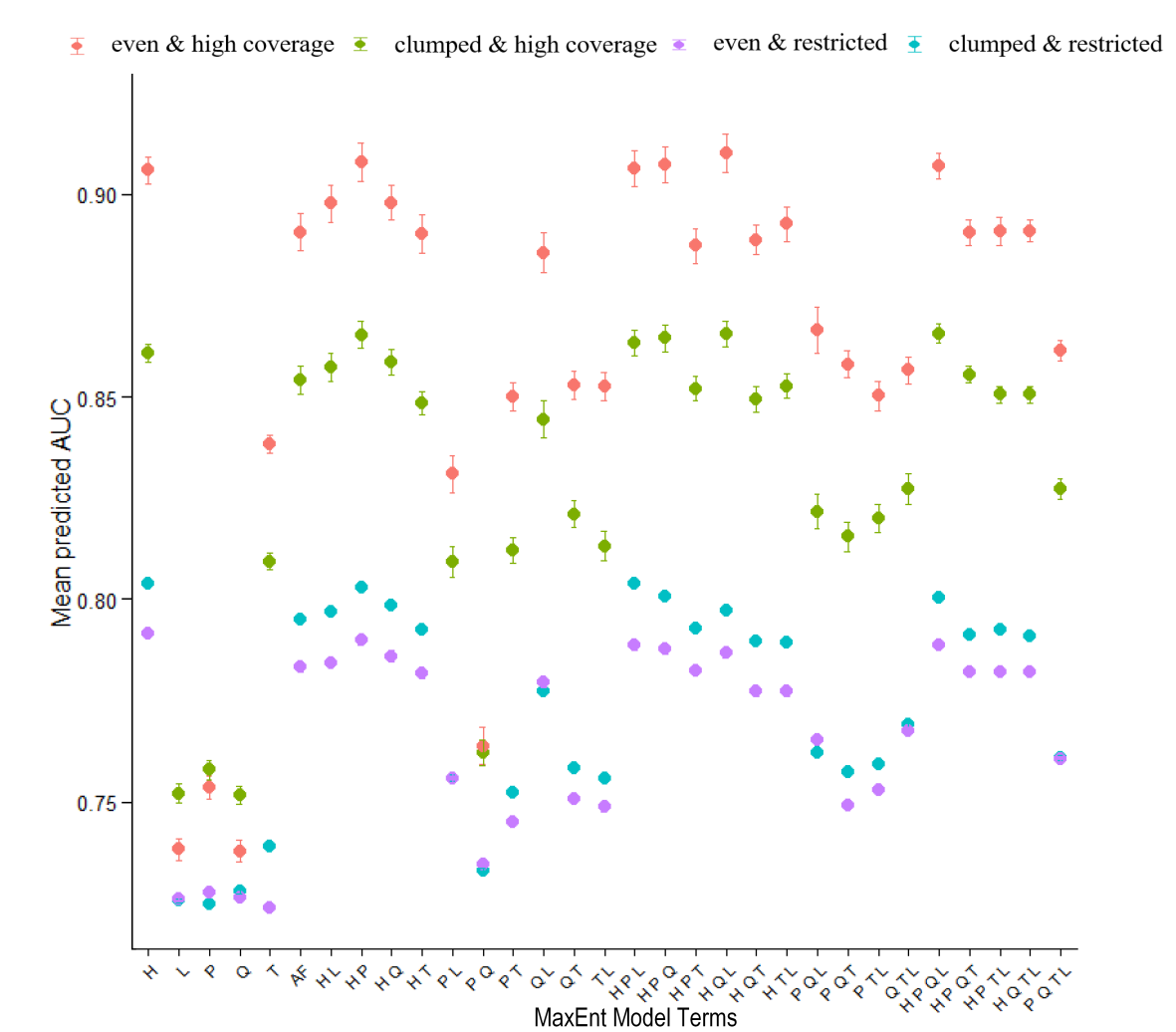

Supplement: S3 Fig — Letter labels on x-axis represent model terms (Hinge—H, Product—P, Quadratic—Q, Threshold—T, Linear—L, Auto Feature—AF). Points represent mean AUC score over a set of 5000 simulated species where a prediction of the true range is attempted using a set of simulated sampling points, with whiskers showing the 95% confidence intervals. Different colours show the predictive accuracy of subsets of the 5000 datasets when binning the input samples from each dataset into either high or low clumping and high or low coverage of the simulated “true” range. (TIF) [file pone.0187602.s003.tif]

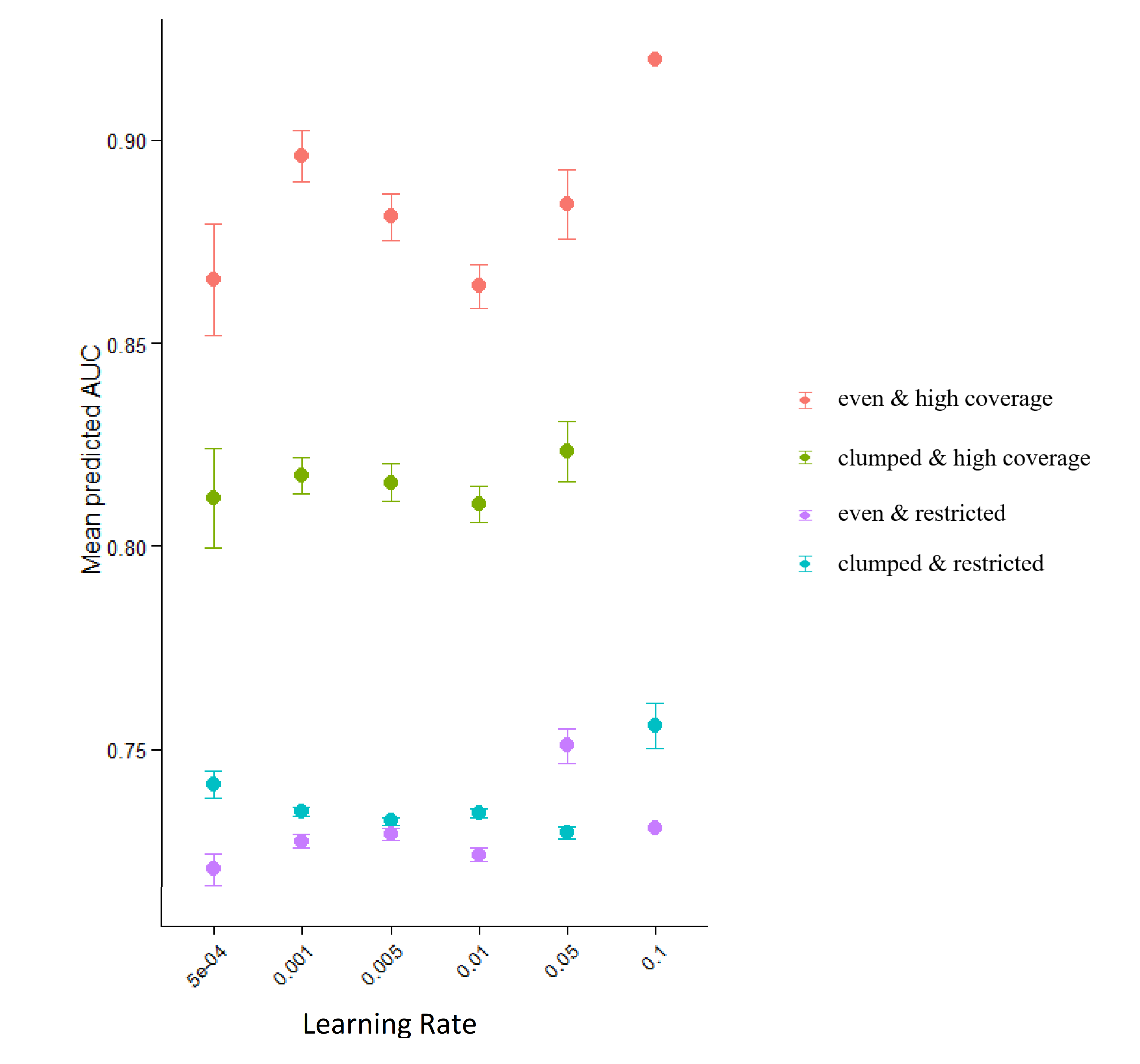

Supplement: S4 Fig — Points represent mean AUC score over a set of 5000 simulated species where a prediction of the true range is attempted using a set of simulated sampling points, with whiskers showing the 95% confidence intervals. Different colours show the predictive accuracy of subsets of the 5000 datasets when binning the input samples from each dataset into either high or low clumping and high or low coverage of the simulated “true” range. (TIF) [file pone.0187602.s004.tif]

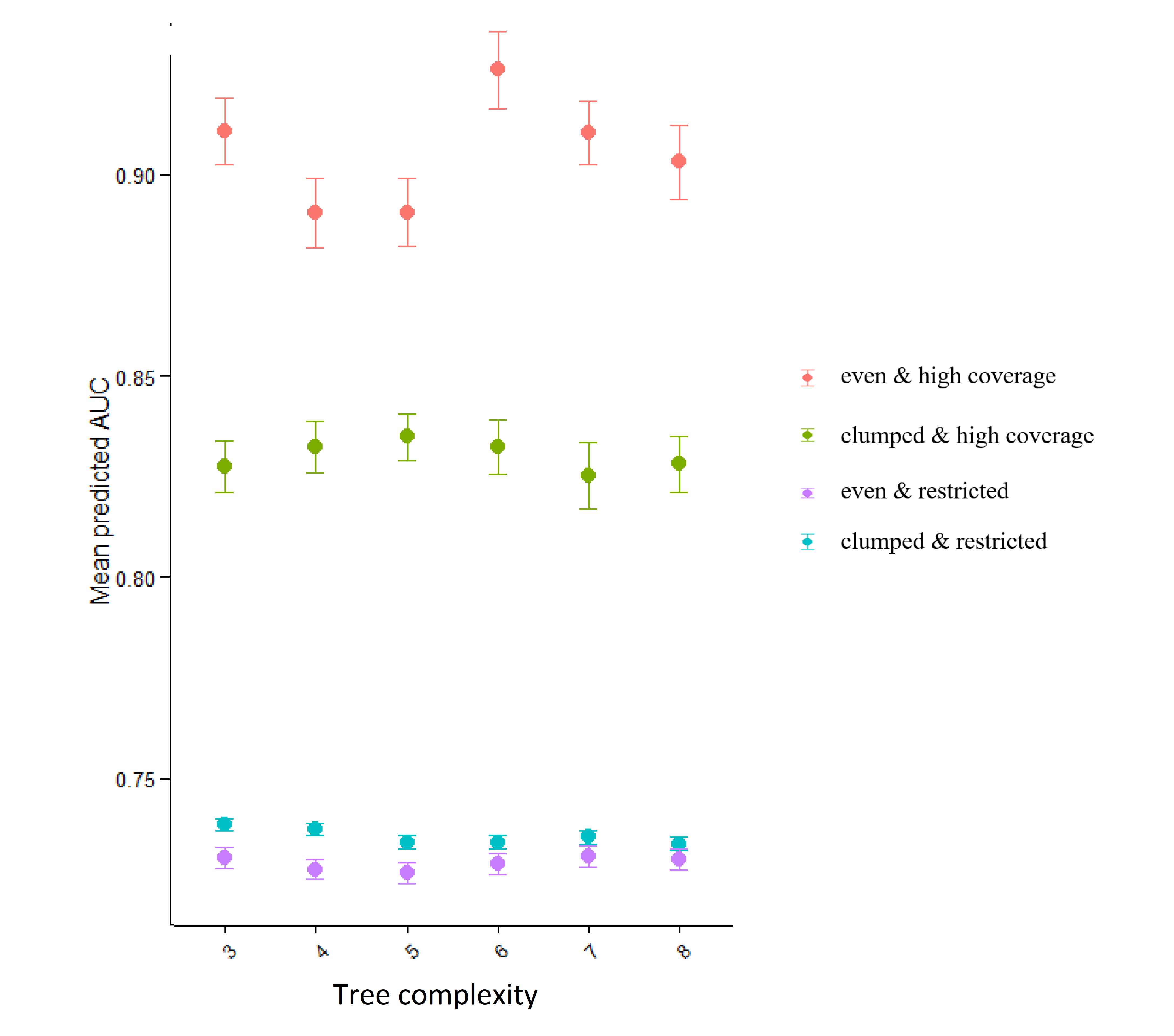

Supplement: S5 Fig — Points represent mean AUC score over a set of 5000 simulated species where a prediction of the true range is attempted using a set of simulated sampling points, with whiskers showing the 95% confidence intervals. Different colours show the predictive accuracy of subsets of the 5000 datasets when binning the input samples from each dataset into either high or low clumping and high or low coverage of the simulated “true” range. (TIF) [file pone.0187602.s005.tif]

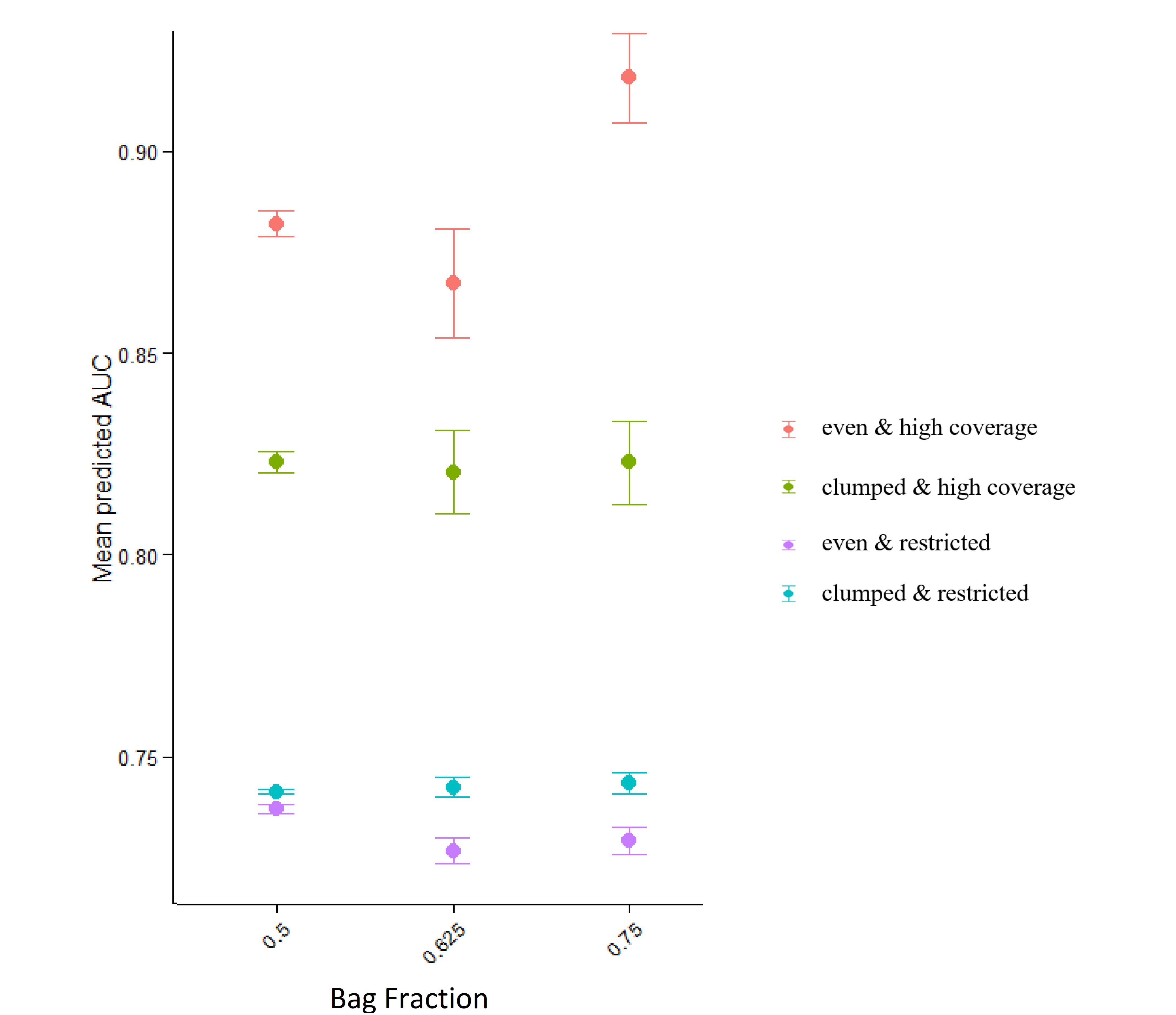

Supplement: S6 Fig — Points represent mean AUC score over a set of 5000 simulated species where a prediction of the true range is attempted using a set of simulated sampling points, with whiskers showing the 95% confidence intervals. Different colours show the predictive accuracy of subsets of the 5000 datasets when binning the input samples from each dataset into either high or low clumping and high or low coverage of the simulated “true” range. (TIF) [file pone.0187602.s006.tif]

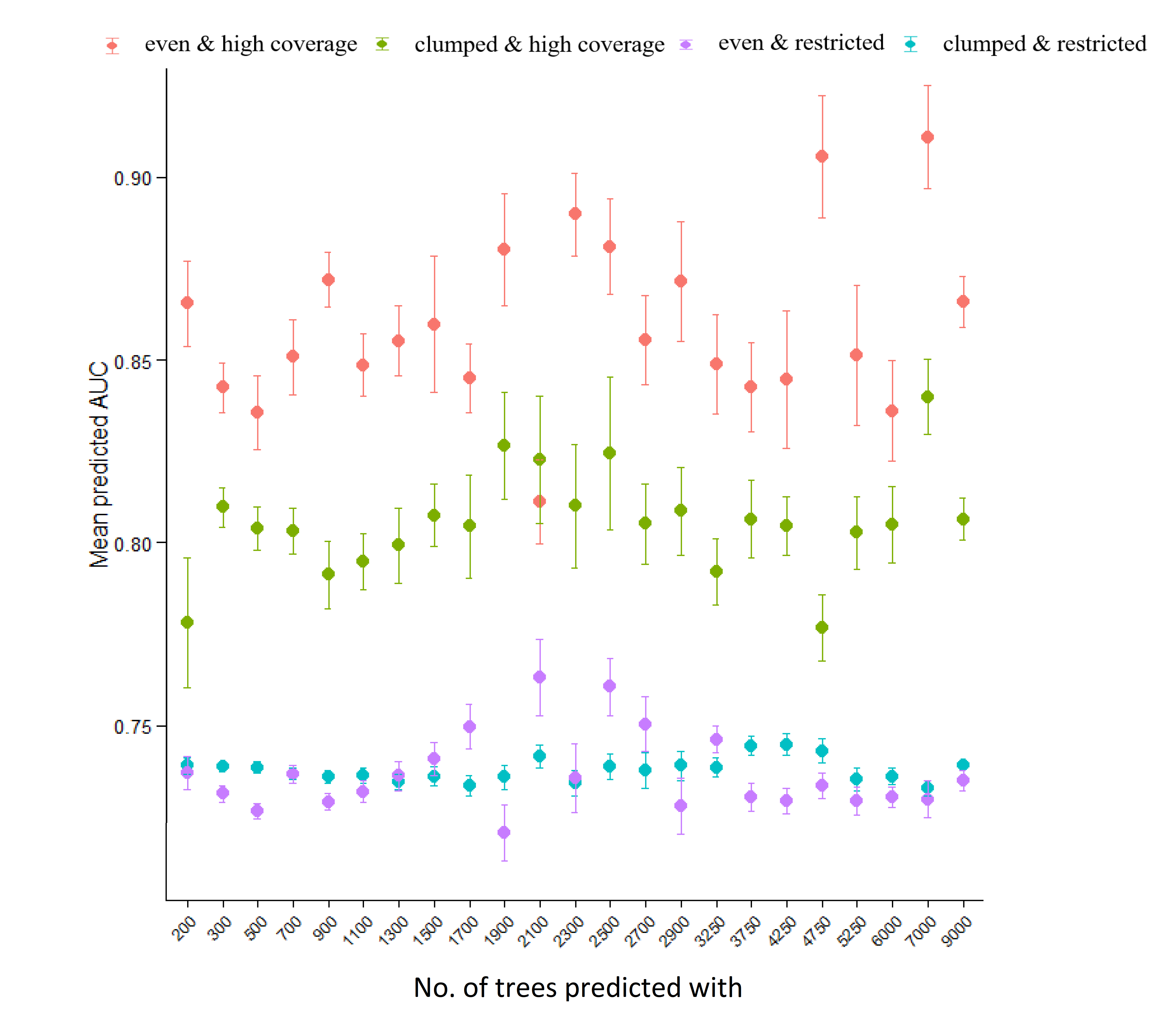

Supplement: S7 Fig — Points represent mean AUC score over a set of 5000 simulated species where a prediction of the true range is attempted using a set of simulated sampling points, with whiskers showing the 95% confidence intervals. Different colours show the predictive accuracy of subsets of the 5000 datasets when binning the input samples from each dataset into either high or low clumping and high or low coverage of the simulated “true” range. (TIF) [file pone.0187602.s007.tif]
