# Supplementary material for: Evaluating Bayesian spatial methods for modelling species distributions with clumped and restricted occurrence data
Source: PLoS One. 2017 Nov 30;12(11):e0187602. doi: 10.1371/journal.pone.0187602 (PMC5708625; doi:10.1371/journal.pone.0187602)
Supplement: S1 Table — Letters in ‘Setting’ columns indicate abbreviations used and numbers previous analyses from the literature. (DOCX) [file pone.0187602.s009.docx]

**Table S1. Options tested for four different species distribution model methods (for details see text).** Letters in ‘Setting’ columns indicate abbreviations used and numbers previous analyses from the literature.

| Method | Setting | DETAILS |
| --- | --- | --- |
| MAXENT | Beta – regularization (BR)([30](#_2250f4o)) | Value of 1 to 20 |
| MAXENT | Model complexity (MC)([30](#_2250f4o)) | Any combination of: nolinear, noquadratic, noproduct, nothreshold, nohinge, noautofeature |
| Spatial Bayesian | Mesh complexity (CO)([23](#_pkwqa1)) | Cut off from 0.5 to 15 |
| BRT | Bag Fraction (BF)([31](#_haapch)) | 0.5, 0.65, 0.75 |
| BRT | Learning rate (LR)([31](#_haapch)) | 0.0001 to 0.1 |
| BRt | Tree complexity (TC)([31](#_haapch)) | 2 to 8 |
| BRT | No. of trees for prediction (NT)([31](#_haapch)) | First 200, first 400, Best guess |
|  |  |  |
| All | Covariate choice (CC)([15](#_xvir7l)) | Most parsimonious model chosen by minimal AIC |
| All | Spatial thinning (ST)([32](#_319y80a)) | Spatially-thinned presence points to reduce autocorrelation |
| All | Spatial weighting of pseudo-absence (SW)([33](#_1gf8i83)) | Spatially-correlated pseudo-absence pts |
| All | Random pseudo-absence (R)([33](#_1gf8i83)) | Random pseudo-absence pts |
